# Morphine Narcosis

**Published:** 1895-05

**Authors:** J. F. Eaves

**Affiliations:** Millican, Texas


For Texas Medical Journal.

### MORPHINE NARCOSIS.

### The Case of the Quadruple Murderer.

BY J. F. EAVES, M. D., MILLICAN, TEXAS.

ON January 30th, I was summoned, by the sheriff of this county to attend a man three miles south of town, suffering from an overdose of sulphate morphia. I reached him at 7:30 a. m., and found that he had taken, the evening before, about 9:30, twenty-four and three-quarters grains of sulphate morphia. He was almost pulseless,—none at the wrist,—his respiration was not perceptible. The pupil was contracte thoroughly, eyelid remained open when pushed back, skin cold, and completely cyanotic. With a Camman's stethescope, I found his heart beat twenty-six per minute, and very weak. I told the sheriff that it was foolish to try to do anything to restore him, as he would be dead in less than an hour, but he (the sheriff) was very anxious that he "be cured," and to satisfy the ardent peace officer, I gave ¼ gr. apomorphine and 30 min. of as heavy solution of the permanganate potash, dissolved in hot water, as my hypodermic syringe would carry. When the needle was introduced, there was no sign of any pain whatever. Noting no change, at 8 a. m. I repeated the permanganate, and then left him to die, and went to breakfast, about three hundred yards away. When I returned, at 9:30 a. m., I found, on introducing the needle, some signs of pain, or muscular reaction. At 10 a. m. the same dose was repeated. At 10:15 the pulse at the wrist was plainly felt, and counted 41 per minute; respiration had returned to a perceptible degree, but too irregular to be counted. At 10:45 the permanganate was repeated, together with ¼ gr. apomorphine. Near 11 o'clock he vomited most of his dinner, eaten the day before. At 11:30 the permanganate was repeated, and at 12.05 p. m., when the same dose was administered, he uttered some incoherent words and caught at the syringe. The same dose, a heavy saturated solution, was then administered every thirty minutes, until about 2 p. m., when Dr. Bell, of Navasota, arrived, and with his assurance that the antidote, which seemed to me to work like magic, would do no harm, he having had experience with the remedy, and after a hasty consultation, we gave hypodermically 180 mins., or a 30-min. hypodermic syringe six times, as fast as we could introduce it. nothing more was then given until 3:30, I having been called away, leaving Dr. Bell, who gave the last of a 210 grain solution.

The sheriff left with the patient in a wagon at 4 o'clock, for Bryan, a distance of twenty miles.

When I arrived that morning, and saw the man's condition, and found from the druggist that he had sold him 25 grains of morphine, and from his relatives, who saw him take the whole of it, read a note confessing having killed all the people at section 12, I thought he would surely die, and felt that it would be better for him and the people.

I think the condition of his stomach prevented the absorption of the drug, possibly, to some extent, for it looks like that amount ought to kill any man in ten hours. I write this article, and hope if any of the profession have occasion to use this antidote they will report it in the JOURNAL, so I will see their opinion, and results. I believe it was there where I first saw it mentioned as an antidote.

